# Managing Known Difficult Airways in Obstetric Patients Using a Flexible Bronchoscope and IRRIS: A Case-Illustrated Guide for Nonexpert Anesthesiologists, without Surgical Backup

**DOI:** 10.1155/2021/6778805

**Published:** 2021-10-08

**Authors:** Kjartan E. Hannig, Rasmus W. Hauritz, Christian Jessen, Jan Herzog, Anders M. Grejs, Michael S. Kristensen

**Affiliations:** ^1^Department of Anesthesiology, Kolding Hospital, Kolding, Denmark; ^2^Department of Anesthesiology, Horsens Hospital, Horsens, Denmark; ^3^Department of Clinical Medicine, Faculty of Health, Aarhus University, Aarhus, Denmark; ^4^Department of Anesthesiology, Odense University Hospital, Odense, Denmark; ^5^Department of Intensive Care Medicine, Aarhus University Hospital, Aarhus, Denmark; ^6^Department of Anesthesiology, Centre of Head and Orthopedics, Rigshospitalet, University Hospital of Copenhagen, Copenhagen, Denmark

## Abstract

Pregnancy is associated with anatomical and physiological changes leading to potential difficult airway management. Some pregnant women have known difficult airways and cannot be intubated even with a hyperangulated videolaryngoscope. If neuraxial techniques are also impossible, awake tracheal intubation with a flexible bronchoscope may be one of the few available options to avoid more invasive techniques. The Infrared Red Intubation System (IRRIS) may help nonexpert anesthesiologists in such situations and may enhance the chance of successful intubation increasing safety for the mother and the fetus, especially in hospitals without the ear, nose, and throat surgical backup.

## 1. Introduction

Pregnancy is associated with anatomical and physiological changes predisposing to difficult airway management, which can be expected from gestation week 20 and until 2 days postpartum [1, 2]. Fluid retention occurs in the entire body including the airway [1]. Edema and vascularity of the upper airway increase resulting in increased risk of airway swelling (and bleeding), which can develop rapidly and may progress even further during labor [1, 2]. The growing uterus displaces the diaphragm cranially, which reduces functional residual capacity (FRC) [1, 2]. The metabolic rate of the pregnant woman increases due to the demands of the fetoplacental unit [1, 2]. This impaired balance between oxygen delivery and consumption explains why pregnant women may desaturate rapidly after induction of general anesthesia, meaning that the safe apnea time is reduced considerably [1, 2]. The risk of aspiration of stomach content increases due to decreased lower esophageal sphincter tone caused by progesterone and the upward displacement of the diaphragm and stomach [1, 2]. Gastric emptying remains normal during pregnancy but is slowed during labor [1].

Guidelines for the management of known difficult airways in nonobstetric patients have been published [3, 4]. For pregnant women, specific guidelines exist both for unexpected and expected difficult airways [1, 2].

Some common features among nonpregnant and pregnant patients in general, where airway management can be predicted to be very difficult and for whom awake tracheal intubation should be considered, are (1) previous difficult airway, (2) significant neck pathology, (3) severely reduced mouth opening, and (4) severely reduced neck movements. Neck pathology from tumor, previous surgery, or radiation increases the risk of failure in every aspect of airway management [4–8]. Mouth opening is normally 40−60 mm depending on gender, height/weight, age, and ethnicity [9]. Presumably, the minimal safe requirements are >30 mm for direct laryngoscopy [10, 11], >20 mm for supraglottic airway device placement [11–13], and >20 mm for hyperangulated videolaryngoscope, depending on the videolaryngoscope and the blade [11, 14, 15]. However, examples of lesser mouth openings with these different techniques have been described [10, 11, 13, 16–18]. Cervical range of motion (ROM) is greatest at the age of 20–30 years (normally about 65° flexion and 75° extension, e.g., ROM 140°) and declines linearly with age (normally about 40° flexion and 50° extension in persons > 80 years, e.g., ROM 90°) [19]. Severe fixed flexion deformity of the neck, especially with an inability to extend, may preclude direct laryngoscopy and sometimes also hyperangulated videolaryngoscope intubation [4, 20]. Therefore, in some cases, intubation with a flexible bronchoscope (FB) may be the only possibility [4, 20].

Psoriasis vulgaris is an inflammatory skin disease, which affects approximately 2-3% of the general population [21]. Approximately 10–20% of patients with psoriasis have psoriatic arthritis, and of those with arthritis, approximately 5% have axial manifestations (psoriatic spondyloarthritis); however, affection of the temporomandibular joint is extremely rare [21].

The present case report describes a patient, in whom both hyperangulated videolaryngoscope intubation and spinal anesthesia had previously been impossible, and anesthesia for cesarean section was required. The discussion focuses on pragmatic tools for nonexpert anesthesiologists who do not perform FB intubation on a regular basis and do not have in-house ear, nose, and throat (ENT) surgical backup.

## 2. Case Presentation

Written informed patient consent for publication was obtained. Approval from Committee on Health Research Ethics was not required according to Danish law.

A 39-year-old woman (height 165 cm; weight 75 kg) presented for elective cesarean section. She had a 13-year history of psoriatic arthritis with severe spondylitis and involvement of the temporomandibular joint. In 2013, the patient underwent subacute cesarean section (category 3) [22]. Spinal anesthesia was difficult and without a sufficient effect. The decision for general anesthesia was made, and a rapid sequence induction was performed. Direct laryngoscopy revealed a Cormack and Lehane score of four, and even with a hyperangulated videolaryngoscope (McGrath–Series 5, Aircraft Medical, Edinburgh, UK), intubation was impossible. The surgical procedure was thus performed with a facemask and the patient breathing spontaneously on sevoflurane. In 2019, a minor elective gynecological procedure again necessitated anesthesia. Spinal anesthesia was attempted once again, but despite multiple attempts by two anesthesiologists, it proved to be impossible. The procedure was instead performed under surgically placed local anesthesia and propofol sedation on spontaneous breathing.

**Figure 1:**
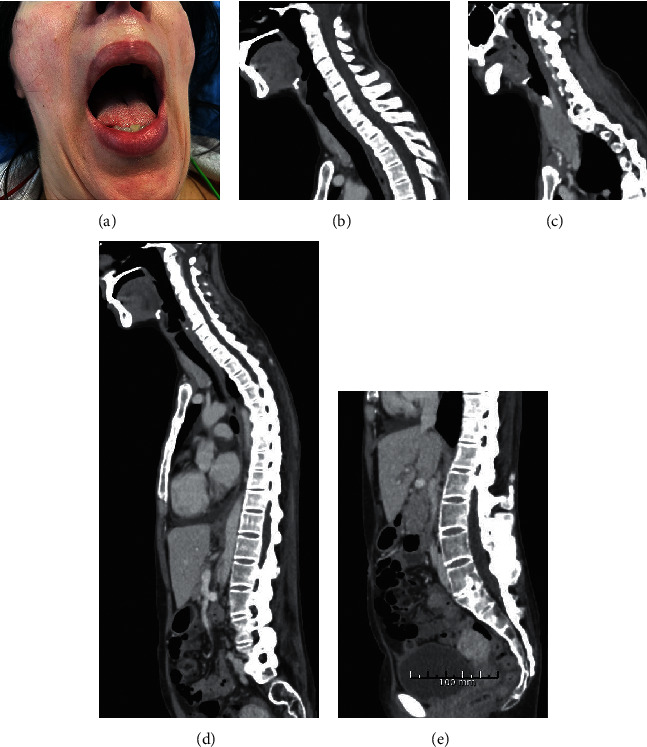
The patient demonstrating mouth opening of 25 mm (a). CT scan of the cervical column showing the midline view with block vertebrae C2/C3 and C5/C6 (b) and paramedian view showing fusion of facet joints C2–C6 (c). Ossification of the anterior longitudinal ligament with anterior bridging osteophytes seen in the thoracic region (d) and fusion of all spinous processes in the lumbar region (e).

Airway examination preoperatively revealed a Simplified Airway Risk Index (SARI) score of 8. Mouth opening was 25 mm (Figure 1). Neck movements were severely limited, and the head could not be extended from the neutral position (0°), but flexion could be performed (about 30–40°). The modified Mallampati score was 3, the thyromental distance was 6–6.5 cm, and the patient was unable to prognath. The cricothyroid membrane was easy to palpate. A computed tomography (CT) scan from 2020 showed block vertebrae C2/C3 and C5/C6 and fusion of facet joints C2–C6 in the cervical region and fusion of multiple facet joints and fusion of all spinous processes in the lumbar region (Figure 1).

Needle-through-needle combined spinal-epidural anesthesia proved impossible, even though attempts were made by two consultants on multiple levels, using both median and paramedian techniques and with ultrasound guidance.

The patient was placed in an upright sitting position, supplementary oxygen by nasal cannula 3 L/minute was provided, and propofol and remifentanil infusions were initiated (0.4–0.6 *μ*g/kg/minute and 0.03–0.05 *μ*g/kg/minute, respectively). Glycopyrrolate 0.2 mg IV (Meda, Solna, Sweden) and ondansetron 4 mg IV (Accord, Solna, Sweden) were administered. At all times, the patient was alert and oriented, able to respond in short sentences, and had sufficient respiration with peripheral saturations above 99%. Topicalization was performed with lidocaine 2% with 5 *μ*g/ml epinephrine (Amgros, Copenhagen, Denmark). A different formulation was used for spray application only, which was lidocaine 10% (Xylocaine Pump Spray 100 mg/ml, AstraZeneca, Södertälje, Sweden). Inhalation (4 ml ×2), transtracheal injection (2 ml ×1), spray application (3 puffs ×3), and for potential backup, nasal route application with a mucosal atomization device (MAD Nasal, Teleflex, Waine, PA, USA) and a local anesthetic soaked ribbon gauze (1 ml ×1, respectively, 3 ml ×1) were applied. Intubation was performed with an FB (Ambu aScope^TM^ Regular, Ambu A/S, Ballerup, Denmark), an LMA Fastrach^TM^ endotracheal tube with an internal diameter of 6.5 mm (Teleflex, Beaconsfield, UK), a Berman intubating airway (Vital Signs, Totwa, NJ, USA), and the Infrared Red Intubation System (IRRIS, Guide In Medical, Nazareth, Israel). The procedure was straightforward, and the intubation itself took less than 2 minutes (Video 1). Visual confirmation of tube placement, gentle cuff inflation, and immediate capnography were followed by induction with propofol 100 mg IV and sevoflurane for maintenance of anesthesia. After delivery of a baby girl (Apgar scores of 8/10), this was changed to propofol and remifentanil infusions.

## 3. Discussion

Delivery in obstetric patients with known difficult airways optimally involves a team consisting of an expert anesthesiologist skilled in awake tracheal intubation with a FB, an obstetric anesthesiologist skilled in ultrasound-guided neuraxial blockade, a senior obstetrician, a midwife, a pediatrician/neonatologist, and an ENT surgeon [2]. Primary and back up plans should be discussed in advance (flowchart 1). Early elective cesarean section in week 38 (instead of week 39) can be planned primarily to minimize the risk of the patient going into labor, accepting a slightly increased risk of respiratory complications for the newborn [23]. In case of early labor, the patient should be instructed to present at the hospital at an early stage. The distance to the hospital should be considered. If the distance is long, the patient may present late, giving little time for anesthetic management (even though every medical specialty is present). If the distance is short, there is potentially ample time for safe anesthetic management (even though, e.g., ENT surgical backup may be lacking). In this case, it was deemed unsafe to let the patient proceed with a natural birth, so a category 3 cesarean section [22] would have been used in case of early labor.

In elective cesarean section, neuraxial techniques can avoid the need for airway management [1, 2, 4], and the use of ultrasound may enhance the chances of success [2, 24]. As both single-shot spinal and epidural top-up can fail, a combined spinal-epidural is likely to provide the greatest probability for success. This can be placed at the L3/4 level (or L2/3), and a full spinal dose can be administered (e.g., bupivacaine heavy 10–12 mg + fentanyl or sufentanil). If a bilateral T4 sensory level is not reached within 20–30 minutes, a carefully titrated epidural top-up with, e.g., lidocaine 2% + epinephrine 5 *μ*g/ml (consider adding bicarbonate) can be administered, e.g., initially 2-3 ml test-dose followed by 3–5 ml at 4-5-minute intervals. The most important aspect is through testing (including dermatomal level) and extreme caution to identify and avoid a high block [1].

Some patients cannot be intubated with a hyperangulated videolaryngoscope [4, 8, 20], and in case of failed (or impossible) neuraxial anesthesia, awake tracheal intubation with a FB is the primary choice if rescue invasive techniques are to be avoided [4, 8]. The advantages of an awake patient are that a patent airway is preserved (with the largest possible airway diameter due to preserved intrinsic airway muscle tone), spontaneous breathing is preserved (hence oxygenation), the glottic opening is easier to localize (air bubbles) and easier to intubate (naturally aligned oropharyngeal axis), the patient can be sitting (thus avoiding aortocaval compression), and there is some protection against aspiration [4, 5]. Since the introduction of videolaryngoscopes in 2001, awake tracheal intubation with a FB may have become an underutilized technique [5, 6]. This may be due to lack of confidence in skills, reluctance due to concerns regarding patient discomfort, and time consumption. Most patients, however, do not perceive this as uncomfortable [4, 25, 26], and the median time to perform awake tracheal intubation with a FB is only 8 minutes longer than for tracheal intubation after induction [5, 26, 27].

The patient should be placed in an upright sitting position and supplementary oxygen should be administered. The cricothyroid membrane should be identified and properly marked if necessary, with the aid of ultrasound [4, 5, 28].

No regime for sedation and topicalization has been found superior in nonpregnant patients [3, 4, 29]. Sedation should be titrated carefully and in small doses corresponding to a Ramsay sedation scale of 2 (cooperative, oriented, and tranquil). Hypnotic drugs and opioids (e.g., propofol, ketamine, midazolam, sevoflurane, fentanyl, and remifentanil) can cross the placenta and may have a depressing effect on the newborn. Hence, shorter acting agents (or agents with antidotes) and sevoflurane may be preferred during antenatal care [1]. If delivery is expedient after the initiation of sevoflurane maintenance, there is likely to be limited time for uptake and distribution into either the mother or the fetus; however, anticipation of respiratory depression of the newborn and assisted ventilation after delivery can ensure excretion of the anesthetic gas. After delivery, maintenance of anesthesia can be achieved with propofol and remifentanil, which decreases the likelihood of postoperative nausea and vomiting (PONV) and is beneficial if the uterus does not contract sufficiently [1]. Optimal topicalization is the key to success. Local anesthetics with added epinephrine can be used, reducing the risk of mucosal bleeding. Presumably, the risk of negative impact on uterine blood flow in this setting is negligible [22], and either way safe airway management of the mother without airway bleeding is the first priority. The maximal dose for topical lidocaine is 9 mg/kg [3, 4].

Oral intubation is the preferred route for pregnant women to avoid the risk of epistaxis which would impair the view and potentially complicate an already difficult intubation [1, 5]. The rate of possible tube impingement during FB is reduced, if specialized tubes are used (e.g., LMA-Fastrach^TM^ ETT or Parker Flex-Tip^TM^ tracheal tube), the opening of the bevel is oriented posteriorly, and the gap between the tube and the FB is minimized [3, 30].

IRRIS can potentially enhance the success rate of awake tracheal intubation with FB. It seems that not only nonexperts who only occasionally perform this procedure [5, 31] but also expert anesthesiologist benefit substantially from this [32]. The idea of retrograde light-guided laryngoscopy is not new. Previously, a method with direct laryngoscopy and a flashlight held on the front of the neck has been published [33, 34]. IRRIS is a small device placed on the skin on the cricothyroid membrane [31]. It emits infrared blinking light, which penetrates the tissue; it is invisible to the naked eye but results in a visible bright blinking light on the video monitor screen of videolaryngoscopes and FBs that do not have infrared filters [31]. IRRIS has been shown to be safe and beneficial in the elective videolaryngoscope intubation of lean patients with normal airways [35], elective videolaryngoscope intubation of extreme obese patients [36], and elective awake tracheal intubation with FB in patients with known difficult airways [31, 32]. The light emerging from the trachea facilitates the identification of the glottis and may be a tool that will make it easier to intubate the most difficult airways with pathology and distortion [31, 32].

If awake tracheal intubation with a FB fails, potential backup plans could fall into the following 4 categories:

1. If no eminent threat to the mother or fetus exists (and the patient is not in labor), the procedure can be stopped. The patient can then be immediately transferred to a hospital with higher levels of airway expertise (e.g., FB and ENT surgical backup).
2. Awake surgical tracheostomy (if an ENT surgeon is present), awake percutaneous dilatational tracheostomy (if a specially trained intensivist is present), or awake cricothyrotomy (performed by an anesthesiologist if neither ENT surgeon nor intensivist is present).
3. Deep sedation/general anesthesia with preserved spontaneous ventilation (e.g., sevoflurane, ketamine, or TIVA induction) or induction of anesthesia with apnea, full relaxation, and controlled ventilation [5]. Both potentially carry a significant risk of failure [4] and which one is chosen must be decided on a case-by-case basis [2]. After failed awake tracheal intubation with FB, the airway must be presumed to be at least partially topicalized. If facemask ventilation has been attempted and found to be difficult/impossible, immediate 2nd generation supraglottic airway device placement should be performed [1]. Depending on the circumstances, a single intubation attempt with a hyperangulated videolaryngoscope by the most experienced practitioner can be considered.
4. Emergency front of neck access (eFONA) under general anesthesia and full relaxation [4]. This should be avoided at all costs since it has demonstrated a failure rate of more than 50% when performed by anesthesiologists on acute (nonobstetric) patients [6]. Preanesthetic identification and marking of the cricothyroid membrane is likely to enhance the success rate of cricothyrotomy [28] and should always be performed in at-risk patients before any airway troubles arise [4, 28].

## 4. Conclusion

In obstetric patients with known difficult airways not manageable with a hyperangulated videolaryngoscope and where neuraxial techniques are also impossible, awake tracheal intubation with a FB may be one of the best options to avoid more invasive rescue techniques.

IRRIS may enhance the chances of success and may be especially beneficial if the procedure has to be performed by nonexpert anesthesiologists (with regard to FB) without ENT surgical backup.

FB intubation skills are preferable in hospitals caring for obstetric patients and optimally available around the clock.

## Data Availability

No data were used to support this case report.

## Supplementary Materials

Supplementary Materials Video 1. Awake tracheal intubation of the patient with a flexible bronchoscope and the Infrared Red Intubation System.

## References

[1] Mushambi M. C., Kinsella S. M., Popat M. (2015). Obstetric Anaesthetists’ association and difficult airway society guidelines for the management of difficult and failed tracheal intubation in obstetrics. *Anaesthesia*.

[2] Mushambi M. C., Athanassoglou V., Kinsella S. M. (2020). Anticipated difficult airway during obstetric general anaesthesia: narrative literature review and management recommendations. *Anaesthesia*.

[3] Ahmad I., El‐Boghdadly K., Bhagrath R. (2020). Difficult Airway Society guidelines for awake tracheal intubation (ATI) in adults. *Anaesthesia*.

[4] Law J. A., Duggan L. V., Asselin M. (2021). Canadian airway focus group updated consensus-based recommendations for management of the difficult airway: part 2. Planning and implementing safe management of the patient with an anticipated difficult airway. *Canadian Journal of Anesthesia/Journal canadien d’anesthésie*.

[5] Aziz M. F., Kristensen M. S. (2019). From variance to guidance for awake tracheal intubation. *Anaesthesia*.

[6] Cook T. M., Woodall N., Frerk C. (2011). Major complications of airway management in the UK: results of the fourth national audit project of the royal college of anaesthetists and the difficult airway society. Part 1: anaesthesia †. *British Journal of Anaesthesia*.

[7] Aziz M. F., Healy D., Kheterpal S., Fu R. F., Dillman D., Brambrink A. M. (2011). Routine clinical practice effectiveness of the Glidescope in difficult airway management. *Anesthesiology*.

[8] Hannig K. E., Hauritz R. W., Jessen C., Grejs A. M. (2019). Acute awake fiberoptic intubation in the ICU in a patient with limited mouth opening and hypoxemic acute respiratory failure. *Case Reports in Anesthesiology*.

[9] Li X.-Y., Jia C., Zhang Z.-C. (2017). The normal range of maximum mouth opening and its correlation with height or weight in the young adult Chinese population. *Journal of Dental Science*.

[10] Aiello G., Metcalf I. (1992). Anaesthetic implications of temporomandibular joint disease. *Canadian Journal of Anaesthesia*.

[11] Crowley S. M., Dalton A. J. (2014). Predicting the difficult airway. *Continuing Education in Anaesthesia, Critical Care & Pain*.

[12] Pennant J. H., White P. F. (1993). The laryngeal mask airway. Its uses in Anesthesiology. *Anesthesiology*.

[13] Maltby J. R., Loken R. G., Beriault M. T., Archer D. P. (1995). Laryngeal mask airway with mouth opening less than 20 mm. *Canadian Journal of Anaesthesia*.

[14] Lawrence M., Ball D., Braga A., Hotvedt G., Rodney G. (2014). Trismus and the limits of laryngoscopy. *Anaesthesia*.

[15] Sanchez R., Añez S. C., Ivars P. C., Santos M. L., Serrano G. V., Camps V. M. (2011). Comparative study of three videolaryngoscopes for nasotracheal intubation with restricted mouth opening: a manikin study. *European Journal of Anaesthesiology*.

[16] Coupe M. H., Johnson D., Seigne P., Hamlin B. (2013). Airway management in reconstructive surgery for noma (cancrum oris). *Anesthesia & Analgesia*.

[17] Patil A. R., Kulkarni K. R., Patil R. S., Madanaik S. S. (2015). Truview PCD-video laryngoscope aided nasotracheal intubation in case series of orofacial malignancy with limited mouth opening. *Journal of Anaesthesiology, Clinical Pharmacology*.

[18] Xue F. S., Yang Q. Y., He N., Xu Y. C. (2008). The modified ventilating tube changer to facilitate tracheal intubation using the GlideScope in patients with a limited mouth opening. *British Journal of Anaesthesia*.

[19] Thoomes-De Graaf M., Thoomes E., Fernández-De-Las-Peñas C., Plaza-Manzano G., Cleland J. A. (2020). Normative values of cervical range of motion for both children and adults: a systematic review. *Musculoskeletal Science and Practice*.

[20] Lai H. Y., Chen I. H., Chen A., Hwang F. Y., Lee Y. (2006). The use of the GlideScope for tracheal intubation in patients with ankylosing spondylitis. *British Journal of Anaesthesia*.

[21] Sieper J., Rudwaleit M., Khan M. A., Braun J. (2006). Concepts and epidemiology of spondyloarthritis. *Best Practice & Research Clinical Rheumatology*.

[22] Lee A., Ngan Kee W. (2019). Effects of vasoactive medications and maternal positioning during cesarean delivery on maternal hemodynamics and neonatal acid-base status. *Clinics in Perinatology*.

[23] Glavind J., Uldbjerg N. (2015). Elective cesarean delivery at 38 and 39 weeks. *Current Opinion in Obstetrics and Gynecology*.

[24] Perlas A., Chaparro L. E., Chin K. J. (2016). Lumbar neuraxial ultrasound for spinal and epidural anesthesia. *Regional Anesthesia and Pain Medicine*.

[25] Schnack D. T., Kristensen M. S., Rasmussen L. S. (2011). Patientsʼ experience of awake versus anaesthetised orotracheal intubation: a controlled study. *European Journal of Anaesthesiology*.

[26] Hannig K. E., Jessen C., Hauritz R. W., Grejs A. M. (2018). Awake fiberoptic intubation in fast track ambulatory surgery: a case report. *A&A Practice*.

[27] Joseph T. T., Gal J. S., DeMaria S., Lin H.-M., Levine A. I., Hyman J. B. (2016). A retrospective study of success, failure, and time needed to perform awake intubation. *Anesthesiology*.

[28] Kristensen M. S., Teoh W. H. (2021). Ultrasound identification of the cricothyroid membrane: the new standard in preparing for front-of-neck airway access. *British Journal of Anaesthesia*.

[29] Cabrini L., Baiardo Redaelli M., Ball L. (2019). Awake fiberoptic intubation protocols in the operating room for anticipated difficult airway. *Anesthesia & Analgesia*.

[30] Kristensen M. S. (2003). The parker flex-tip tube versus a standard tube for fiberoptic orotracheal intubation. *Anesthesiology*.

[31] Kristensen M. S., Fried E., Biro P. (2018). Infrared Red Intubation System (IRRIS) guided flexile videoscope assisted difficult airway management. *Acta Anaesthesiologica Scandinavica*.

[32] Jauho K. R., Johannsen M. L., Hesselfeldt R. T., Kristensen M. S. (2021). Infrared flashing light through the cricothyroid membrane to guide flexible bronchoscopic tracheal intubation. *Anaesthesia Reports*.

[33] Hudson J., Vu M., Vu E. (2010). Successful intubation using retrograde trans-tracheal illumination after laryngoscope light source failure. *British Journal of Anaesthesia*.

[34] Yang T., Hou J., Li J. (2013). Retrograde light-guided laryngoscopy for tracheal intubation. *Anesthesiology*.

[35] Biro P., Fried E., Schlaepfer M., Kristensen M. S. (2018). A new retrograde transillumination technique for videolaryngoscopic tracheal intubation. *Anaesthesia*.

[36] Godoroja D. D., Copaescu C. A., Agache M. C., Biro P. (2019). Impact of retrograde transillumination while securing the airway in obese patients undergoing bariatric surgery. *Journal of Clinical Monitoring and Computing*.

