# Supplementary material for: Managing Known Difficult Airways in Obstetric Patients Using a Flexible Bronchoscope and IRRIS: A Case-Illustrated Guide for Nonexpert Anesthesiologists, without Surgical Backup
Source: Case Rep Anesthesiol. 2021 Oct 8;2021:6778805. doi: 10.1155/2021/6778805 (PMC8519668; doi:10.1155/2021/6778805)
Supplement: Supplementary Materials — Video 1. Awake tracheal intubation of the patient with a flexible bronchoscope and the Infrared Red Intubation System. [file 6778805.f1.docx]

https://drive.google.com/file/d/1AmBOexWWaH9ac2wAI0Sq-_U54FgxJvJ-/view?usp=sharing
